# Electrical Vagus Nerve Stimulation Attenuates Systemic Inflammation and Improves Survival in a Rat Heatstroke Model

**DOI:** 10.1371/journal.pone.0056728

**Published:** 2013-02-12

**Authors:** Kazuma Yamakawa, Naoya Matsumoto, Yukio Imamura, Takashi Muroya, Tomoki Yamada, Junichiro Nakagawa, Junya Shimazaki, Hiroshi Ogura, Yasuyuki Kuwagata, Takeshi Shimazu

**Affiliations:** 1 Department of Traumatology and Acute Critical Medicine, Osaka University Graduate School of Medicine, Osaka, Japan; 2 Unit for Liveable Cities, Human Health Science, Kyoto University Graduate School of Medicine, Kyoto, Japan; University of Pecs Medical School, Hungary

## Abstract

This study was performed to gain insights into novel therapeutic approaches for the treatment of heatstroke. The central nervous system regulates peripheral immune responses via the vagus nerve, the primary neural component of the cholinergic anti-inflammatory pathway. Electrical vagus nerve stimulation (VNS) reportedly suppresses pro-inflammatory cytokine release in several models of inflammatory disease. Here, we evaluated whether electrical VNS attenuates severe heatstroke, which induces a systemic inflammatory response. Anesthetized rats were subjected to heat stress (41.5°C for 30 minutes) with/without electrical VNS. In the VNS-treated group, the cervical vagus nerve was stimulated with constant voltage (10 V, 2 ms, 5 Hz) for 20 minutes immediately after completion of heat stress. Sham-operated animals underwent the same procedure without stimulation under a normothermic condition. Seven-day mortality improved significantly in the VNS-treated group versus control group. Electrical VNS significantly suppressed induction of pro-inflammatory cytokines such as tumor necrosis factor-α and interleukin-6 in the serum 6 hours after heat stress. Simultaneously, the increase of soluble thrombomodulin and E-selectin following heat stress was also suppressed by VNS treatment, suggesting its protective effect on endothelium. Immunohistochemical analysis using tissue preparations obtained 6 hours after heat stress revealed that VNS treatment attenuated infiltration of inflammatory (CD11b-positive) cells in lung and spleen. Interestingly, most cells with increased CD11b positivity in response to heat stress did not express α7 nicotinic acetylcholine receptor in the spleen. These data indicate that electrical VNS modulated cholinergic anti-inflammatory pathway abnormalities induced by heat stress, and this protective effect was associated with improved mortality. These findings may provide a novel therapeutic strategy to combat severe heatstroke in the critical care setting.

## Introduction

During the summer season, heat waves are responsible for a large number of deaths in various parts of the world. Particularly in Japan, there is a growing concern in regard to prevention and treatment of heatstroke because of restrictions placed on power consumption in the aftermath of the Fukushima nuclear disaster, which followed the Great East Japan Earthquake on March 11, 2011 [1]. Heatstroke is a life-threatening disease characterized by hyperthermia associated with a systemic inflammatory response leading to multiple organ dysfunction or failure, including hemorrhage and necrosis in the brain, lungs, heart, gastrointestinal tract, liver, and kidneys [2], [3]. Although various strategies for the treatment of heatstroke-induced inflammation have been used clinically over the past several decades, the clinical efficacy of therapeutic interventions has been limited. Mortality rate in heatstroke patients over the last 50 years has remained in the 10% to 50% range [4]. Thus, a novel therapeutic strategy other than supportive care is required to improve patient outcome.

Previously, in 2000, Borovikova et al. discovered that the central nervous system (CNS), through the vagus nerve, can modulate the level of circulating tumor necrosis factor (TNF)-α induced by endotoxin [5]. This newly identified mechanism, termed the cholinergic anti-inflammatory pathway, is based on the release of acetylcholine, the principle neurotransmitter of the vagus nerve that inhibits the production of proinflammatory cytokines via its α7 nicotinic acetylcholine receptor (α7nAChR) in resident tissue macrophages [6]. Electrical or pharmacological stimulation of the vagus nerve can modulate immune responses in life-threatening conditions such as hemorrhagic shock [7], [8], ischemia and reperfusion injury [9], [10], or sepsis [11]–[13].

CNS disorders are predominant in heatstroke patients. Cerebral dysfunctions that occur during heatstroke include delirium, convulsion, and coma due to cerebral edema and ischemia [14], [15]. In a rodent model of heatstroke, cellular damage markers are upregulated in the hypothalamus, the essential thermoregulatory center in the brain [16], [17]. Thus, we hypothesized that the vagus nerve will also be affected by heat stress and that the cholinergic anti-inflammatory pathway might play a pivotal role in the pathogenesis of heatstroke, and therefore, we investigated the effect of electrical vagus nerve stimulation (VNS) in a rodent model of heatstroke.

## Materials and Methods

### Animals

Specific pathogen-free male Wistar rats weighing 250 to 300 g were obtained from Nihon SLC (Hamamatsu, Japan) and were allowed free access to food and water. All animal experiments were conducted in accordance with guidelines of the Animal Care and Use Committee of Osaka University Graduate School of Medicine and were approved by that committee. Rats were anesthetized by intraperitoneal injection of sodium pentobarbital (50 mg/kg body weight) (Dainippon Sumitomo Pharma Co., Osaka, Japan).

### Induction of heat stress

Rat rectal temperatures were monitored continuously throughout the induction of heat stress. Anesthetized rats were subjected to environmental heat stress using a temperature control device with a warm blanket (BWT-100A; Bio Research Center, Nagoya, Japan).

**Figure 1:**
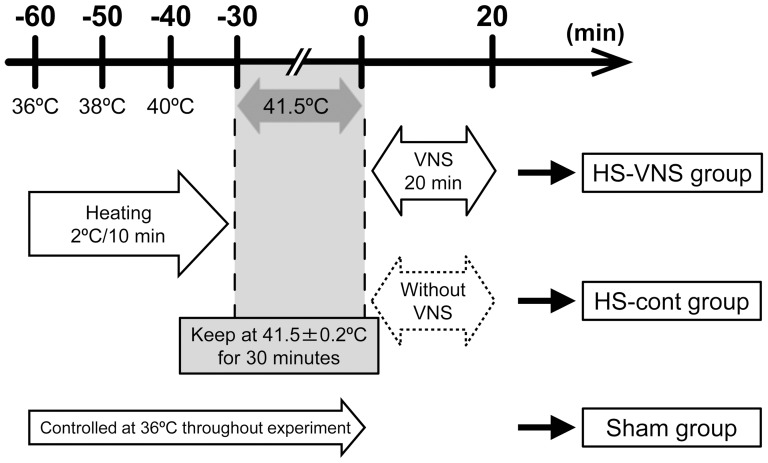
Schema of experimental design and time schedule of heatstroke model.

The time schedule in this experimental study is shown in **Figure 1**. The murine model of heatstroke used in this study has been detailed previously [18]–[20]. Before induction of heat stress, the core temperature of the anesthetized rats was maintained at about 36°C with a heating pad. Next, heat stress was induced by increasing the rectal temperature approximately 1°C every 5 minutes. From the instant the rectal temperature reached 41.5°C, the core temperature was maintained at 41.5 ± 0.2°C for 30 minutes. Then, the heating pad was removed, and the rats were allowed to recover at room temperature (24°C). Finally, saline was injected subcutaneously at a dose of 35 ml/kg to prevent dehydration. The time at which rats were removed from the temperature control device was defined as 0 hours.

### Vagus nerve stimulation

Before induction of the heat stress, a small incision was made to expose the left cervical vagus nerve, and a platinum electrode was then placed under the exposed nerve. Immediately after the completion of heat stress, the platinum electrode was attached to the stimulation device (Master-8 Pulse Stimulator; A.M.P.I., Jerusalem, Israel). Electrical VNS was applied for 20 min at 10 V, 2 ms, and 5 Hz. Following termination of VNS, the electrode was removed, and the incision was closed with interrupted silk sutures.

### Experimental design

Animals were randomly assigned to the sham group, heatstroke without stimulation group (HS-cont group), or VNS-treated heatstroke group (HS-VNS group). The sham group underwent the same experimental procedure but without application of heat stress. Immediately after heat stress, rats in the HS-VNS group received electrical VNS. In the sham group and the HS-cont group, the stimulation electrode was placed immediately under the vagus nerve, but the nerve was not stimulated.

The rats were observed over the 7 days after heat stress in order to calculate survival rates (n  =  23 each in the HS-cont and HS-VNS groups and n  =  8 in the sham group). Separate animals were used for blood sampling and histological analysis. Blood samples were collected at 1, 3, and 6 hours after heat stress (n  =  10 for each time point in the HS-cont and HS-VNS groups and n  =  5 for each time point in the sham group). Perfusion fixation was carried out to prepare tissue specimens at 6 hours after heat stress (n  =  3 each in the sham, HS-cont, and HS-VNS groups, respectively).

### Measurement of serum levels of TNF-α, interleukin-6, soluble thrombomodulin, and soluble E-selectin

The rats were re-anesthetized by intraperitoneal injection of pentobarbital sodium at the allocated time and underwent laparotomy, after which the abdominal aorta was cannulated with a PE50 polypropylene tube (Becton Dickinson Co, Franklin Lakes, NJ). Blood samples were collected through this tube, and the serum was isolated by centrifugation at 3000 x *g* for 15 minutes and frozen at -30°C until measurement.

The serum concentrations of TNF-α, interleukin-6 (IL-6), soluble thrombomodulin (sTM), and soluble E-selectin (sE-selectin) were measured with commercially available ELISA kits (TNF-α and IL-6: R&D Systems, Minneapolis, MN; sTM and E-selectin: Cusabio Biotech, Wuhan, China).

### Histological examinations

#### Tissue fixation

At 6 hours after the completion of heat stress, the rats were anesthetized by intraperitoneal injection of pentobarbital sodium and then perfused transcardially with phosphate-buffered saline (PBS) followed by 4% paraformaldehyde in 0.1 M phosphate buffer (PB). The lung and spleen were dissected, immersed in the same fixative at 4°C for 6 hours, and cryoprotected in a series of sucrose solutions (15%, 20%, and 25% sucrose in 0.1 M PB) at 4°C for 3 days. After the specimens were frozen in OCT compound (Sakura Finetechnical Co. Ltd., Osaka, Japan), they were sliced into 7-µm-thick sections by cryostat (CM3050S; Leica Microsystems, Wetzlar, Germany), and the sections were mounted on slides for staining.

#### Fluorescent immunostaining

The cryosections were blocked by 20% Block Ace (Dainippon Sumitomo Pharma Co., Osaka, Japan) in 0.1 M PB with 0.005% saponin (Sigma-Aldrich Co., St. Louis, MO) and incubated with primary antibodies followed by secondary antibodies overnight at 4°C. The antibodies were dissolved in 0.1 M PB containing 5% Block Ace and 0.005% saponin. The sections were washed with PBS after each reaction. Finally, they were mounted in 0.1 M PB-glycerin (1:1) solution, coverslipped, and observed under a fluorescent microscope (BZ-9000; Keyence Co., Osaka, Japan). The primary antibodies used were 1:100 rabbit anti-rat α7nAChR antibody (H302; Santa Cruz Biotechnology Inc., Santa Cruz, CA), 1:50 rat anti-rat CD11b monoclonal antibody (M1/70; BD Pharmingen, San Diego, CA), and 1:200 mouse anti-rat rat endothelial cell antigen-1 (RECA-1) antibody (ab9774; Abcam Inc., Cambridge, MA). For negative control, nonimmune serum was substituted for primary antibody. All of the secondary antibodies were conjugated to Alexa Fluor (488, 546, and 633: Invitrogen, Carlsbad, CA).

#### Quantification of inflammatory cells and α7nAChR

Quantification of the induction of CD11b-positive cells and the expression of α7nAChR on CD11b-positive cells was performed with National Institutes of Health Image J 1.46 software (NIH, Bethesda, MD). After immunohistochemical double staining with antibodies for CD11b and α7nAChR, counterstaining was performed with DAPI at the time of mounting (AR-6501-01; ImmunoBioScience Corp., Mukilteo, WA). The ratio of the number of CD11b-positive cells to the number of all cells stained by DAPI was calculated in a section from each animal in which five fields were randomly selected and observed at x400 magnification. α7nAChR expression was also evaluated as the percentage of CD11b-positive cells expressing α7nAChR in all CD11b-positive cells by the same calculation method.

### Statistical analysis

Data in the figures are expressed as group means ± standard error of the mean and were analyzed by analysis of variance followed by *post-hoc* Dunnett multiple comparison test. Survival curves were calculated by the Kaplan-Meier method and compared by log-rank test. A *p* value of <0.05 was considered to indicate statistical significance. All statistical analyses were performed with IBM SPSS Statistics version 19.0 for Windows (SPSS Inc., Chicago, IL).

## Results

### Survival assay

**Figure 2:**
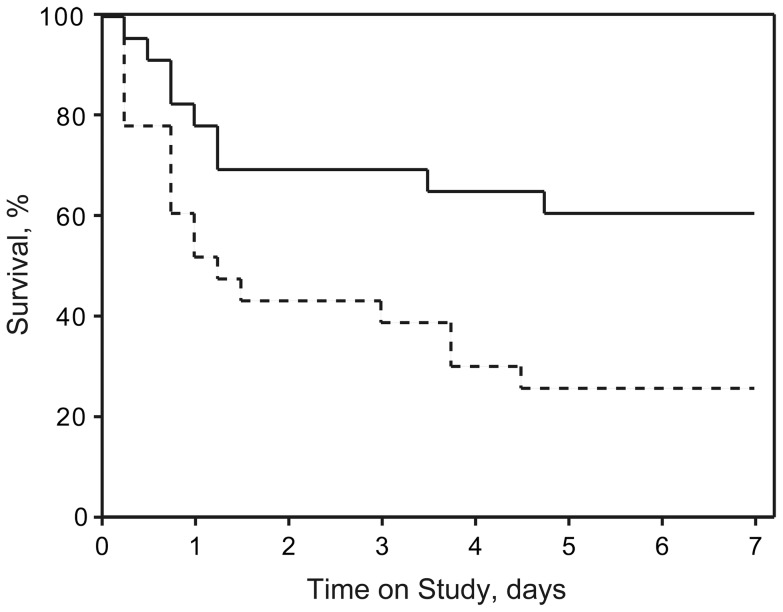
Survival rates during the first 7 days after heat stress. The dotted line represents the survival curve of the rats that underwent heat stress without VNS, and the solid line represents the VNS-treated heatstroke rats (each group, n  =  23). Treatment with VNS was associated with a significantly higher rate of survival against heat stress (*p*  =  0.016). Sham operation caused no mortality in the rats that did not undergo heat stress (n  =  8, data not shown).

Rat survival curves are shown in **Figure 2**. The survival rate at 7 days after heat stress in the HS-cont group was 26% (6 of 23 rats), whereas that in the HS-VNS group was 61% (14 of 23 rats). When compared with the survival rate of heatstroke rats without treatment, VNS-treated heatstroke rats had significantly higher values of percent survival (*p*  =  0.016 by log-rank test).

### Serum levels of TNF-α, IL-6, sTM, and sE-selectin after heat stress

**Figure 3:**
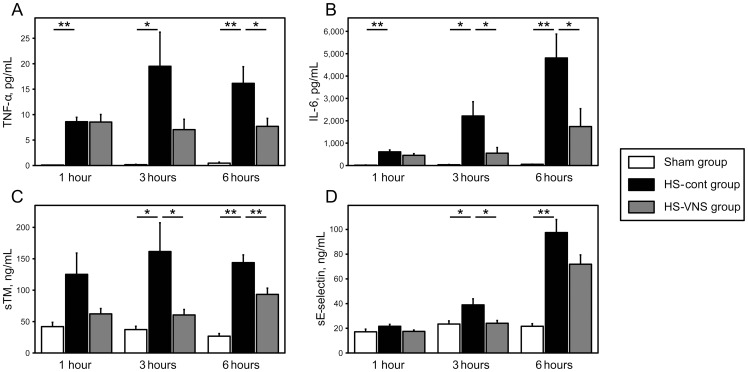
Serial changes in levels of serum TNF-α, IL-6, sTM, and sE-selectin after heat stress. Serum concentrations of TNF-α (**A**), IL-6 (**B**), sTM (**C**), and sE-selectin (**D**) in the sham group, HS-cont group (heatstroke rats without VNS treatment), and HS-VNS group (VNS-treated heatstroke rats) are shown. All data are expressed as mean ± standard error of the mean. n  =  5 at every time point in the sham group and n  =  10 at every time point in the HS-cont and HS-VNS groups. *p <0.05, **p <0.01.

The HS-cont group showed significantly higher levels of TNF-α and IL-6 compared with those in the sham animals at every time point. In the HS-cont group, the mean value of the serum TNF-α level peaked at 3 hours after heat stress and then decreased. Electrical VNS significantly decreased the induction of serum TNF-α level at 6 hours after heat stress (*p* <0.05) (**Figure 3A**). Serum levels of IL-6 in the HS-cont group gradually increased after heat stress and peaked at 6 hours. These elevations were significantly suppressed at 3 and 6 hours after heat stress by VNS treatment (*p* <0.05) (**Figure 3B**).

To evaluate the effects of VNS treatment on endothelium in heat stress, we assessed the serum levels of sTM and E-selectin, the markers for endothelial injury. These markers were significantly higher in the HS-cont group compared with those in the sham group at 3 and 6 hours after heat stress. In the HS-VNS group, the serum level of sTM at 3 and 6 hours was significantly suppressed in comparison with that in the HS-cont group (*p* <0.05) (**Figure 3C**). Similarly, VNS treatment induced a significant decrease in serum level of sE-selectin at 3 hours after heat stress (*p* <0.05) (**Figure 3D**).

### Immunohistochemical detection of α7nAChR expression in CD11b-positive cells in the spleen

**Figure 4:**
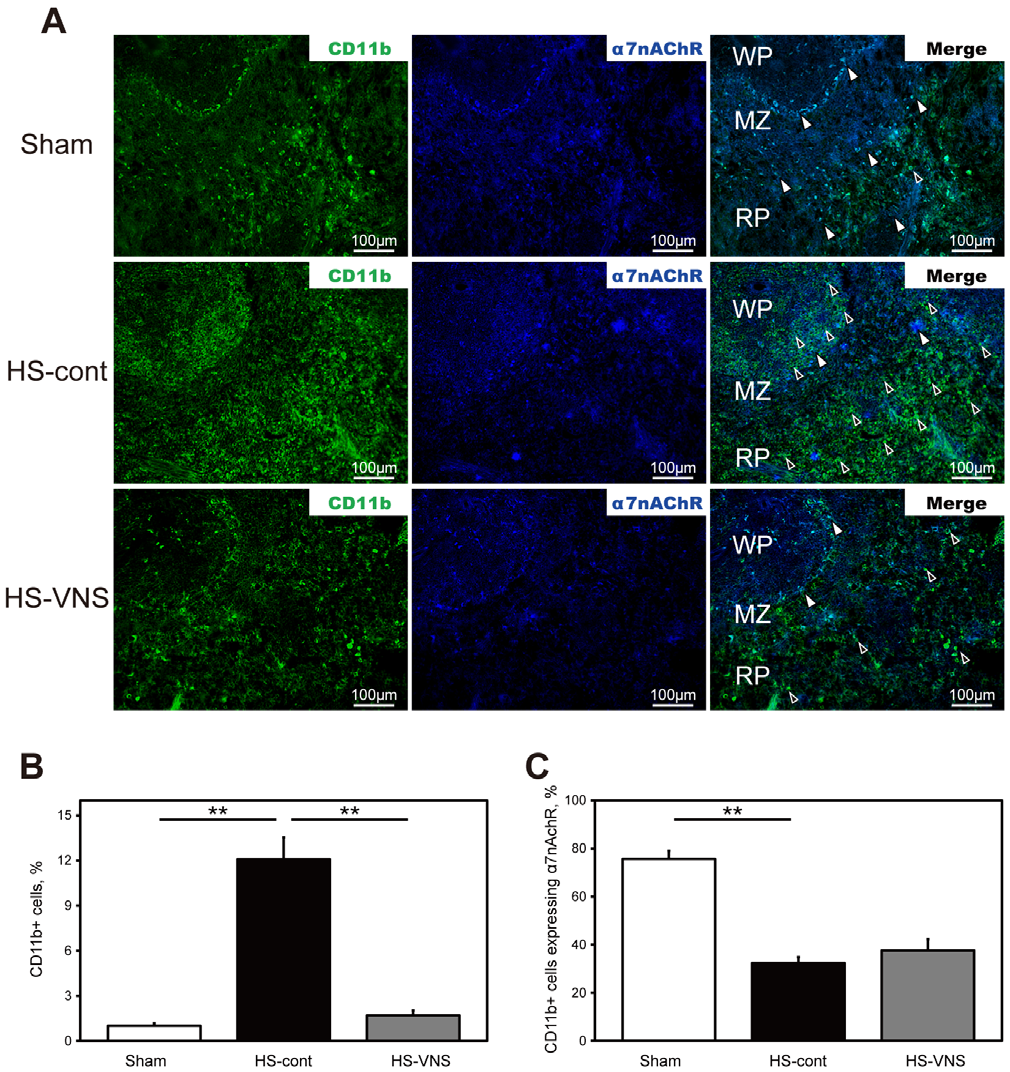
Immunohistochemical expression of CD11b and α7nAChR in the spleen. A) Spleens harvested 6 hours after heat stress were stained for CD11b (green) and α7nAChR (blue). The open arrowheads indicate CD11b-positive cells expressing α7nAChR, and the closed arrowheads indicate CD11b-positive cells without expression of α7nAChR. Bars represent 100 µm, original magnification ×200. MZ, marginal zone; WP, white pulp; RP, red pulp. **B)** Results of statistical analysis for the percentage of CD11b-positive cells in the spleen. **C)** Results of statistical analysis for the percentage of CD11b-positive cells expressing α7nAChR in all CD11b-positive cells. **p <0.01.

To study the inflammatory cell surface expression of α7nAChR immunohistochemically, spleen sections obtained 6 hours after the heat stress were double stained with anti-rat CD11b (a surface marker for inflammatory cells) and anti-α7nAChR antibodies (**Figure 4**). The number of CD11b-positive cells was upregulated in the HS-cont group compared with that in the sham group and was suppressed in the HS-VNS group compared with that in the HS-cont group. Interestingly, we found that most of the CD11b-positive cells induced by heat stress did not express α7nAChR in the spleen.

### Immunohistochemical detection of α7nAChR expression in CD11b-positive cells and endothelial cells in the lung

**Figure 5:**
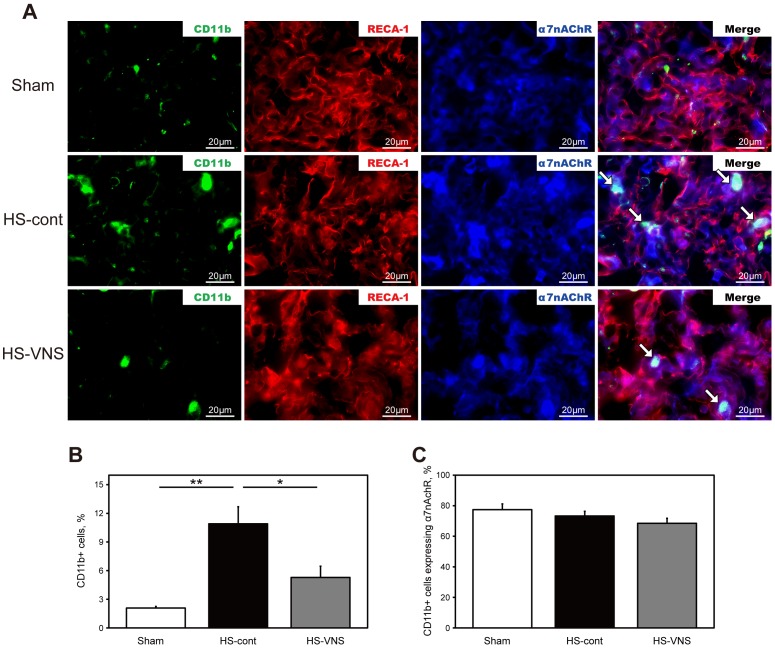
Immunohistochemical expression of CD11b, RECA-1 and α7nAChR in the lung. A) Lungs harvested 6 hours after heat stress were stained for CD11b (green), RECA-1 (red), and α7nAChR (blue). The arrows indicate CD11b-positive cells expressing α7nAChR. Bars represent 20 µm, original magnification ×1200. **B)** Results of statistical analysis for the percentage of CD11b-positive cells in the lung. **C)** Results of statistical analysis for the percentage of CD11b-positive cells expressing α7nAChR in all CD11b-positive cells. *p <0.05, **p <0.01.

To investigate the infiltration of inflammatory cells along with their expression of α7nAChR in heat stress-induced lung injury, lung sections were obtained 6 hours after the heat stress (**Figure 5**). The number of CD11b-positive cells was upregulated in the HS-cont group compared with that in the sham group and was suppressed in the HS-VNS group compared with that in the HS-cont group. Unlike in the spleen, most of the CD11b-positive cells induced by heat stress expressed α7nAChR in the lung.

Next, we estimated alteration in the endothelial expression of α7nAChR. Immunohistochemical double staining was performed with antibodies for α7nAChR and RECA-1, which specifically recognizes rat endothelial cells. Staining revealed that the expression of α7nAChR in the lung endothelial cells also tended to be increased by heat stress.

## Discussion

Heatstroke syndrome is comprised of a wide range of thermoregulatory, inflammatory, coagulatory, immune, and tissue injury responses, and presently, understanding of the endogenous mechanisms is limited. A complex interplay between heat cytotoxicity, systemic inflammatory response, and disseminated intravascular coagulation causes multiple organ dysfunctions. Additionally, brain hyperthermia causes CNS abnormalities, increasing metabolic rate with a reduction in blood flow in the cerebrum [21]. Although some anticoagulant agents such as recombinant activated protein C [22], [23], recombinant thrombomodulin [18], and antithrombin [19] attenuate inflammation and multi-organ system dysfunction in animal experiments, the clinical syndrome of heatstroke is still a challenging problem, and there remains an urgent unmet need for development of new therapeutics. We demonstrated for the first time, to our knowledge, that cholinergic activation by electrical VNS attenuated heat stress-related systemic inflammation, resulting in an increased survival rate in a rat model of heatstroke.

Systemic inflammation plays a pivotal role in the pathogenesis of heatstroke. A variety of cytokines are known to be produced in response to endogenous or environmental heat stress [24]. It is now well accepted that signaling from the vagus nerve has potent anti-inflammatory effects [25]. Electrical VNS against a burn injury model reduced serum levels of TNF-α and IL-6 [26], [27], and in a traumatic brain injury model, the suppressive effect of VNS on TNF-α was shown in intestine [28]. In the present study, we also showed that induction of serum TNF-α and IL-6 was significantly suppressed by electrical VNS treatment in our rat model of heatstroke. In addition, heat stress enhanced the number of inflammatory cells in the lung and the spleen, and VNS treatment hampered it. The obvious and rapid decline in the levels of these mediators by VNS may have contributed to the reduction in multiple organ dysfunctions, resulting in improvement of outcome in the heatstroke rats.

The endothelium is an interactive barrier between blood and tissue that plays a critical role in host immune responses in systemic inflammatory response syndrome. Extreme heat stress directly induces endothelial death by apoptosis [29]. There have been several reports concerning the effect of cholinergic activation against endothelial injury. Acetylcholine and other cholinergic mediators inhibit the expression of E-selectin in endothelial cells stimulated by IL-4 [30]. Another study provided evidence that pharmacologic stimulation of α7nAChR reduces both chemokine production and adhesion molecule expression in endothelium during inflammation [31]. The present study showed that electrical VNS significantly suppressed serum levels of endothelial injury markers sTM and sE-selectin, suggesting that VNS protects endothelium from heat stress. Because widespread hemorrhage, thrombosis, and transmural migration of leukocytes in association with microvascular endothelial injury are prominent features of heatstroke [29], it is challenging to elucidate the regulatory mechanism of the vagus nerve on endothelial cellular function.

α7nAChRs, an important target of the cholinergic anti-inflammatory pathway, are known to be expressed on immune cells, especially macrophages, and endothelial cells [32]. The production of pro-inflammatory cytokines derived from these cells is restricted through stimulation of α7nAChRs [33]. Huston *et al.* [34] reported that electrical VNS failed to inhibit systemic TNF production in splenectomized mice during lethal endotoxemia, indicating that the spleen is a major contributor to the anti-inflammatory effect via the cholinergic pathway. Exposure of inflammatory cells having α7nAChR to acetylcholine in spleen is an essential step to suppress systemic inflammation [6]. To clarify the role of the cholinergic anti-inflammatory pathway in the pathogenesis of heatstroke, we evaluated the expression of α7nAChR on the inflammatory cells. Most of the infiltrated CD11b-postive cells expressed α7nAChR in the lung and not in the spleen. The spleen stores undifferentiated monocytes readily recruitable to augment inflammation at distant sites. After the release of splenic monocytes, they become biologically active when recruited to inflamed organs [35]. It is possible that the enhanced expression of α7nAChR on infiltrated inflammatory cells and endothelial cells in the lung indicates a compensatory reaction to maintain immune homeostasis. However, heat stress would disturb the neural output signal to stimulate α7nAChR and consequently abolish this intrinsic immunomodulatory mechanism. In addition, the infiltrated inflammatory cells in spleen would be held in an immature state and might be unable to produce substantial α7nAChR in the acute phase of heatstroke. Further investigation is necessary to support our supposition.

Lastly, in the present study, electrical VNS treatment was conducted immediately after the heat stress. The time schedule of treatment in this experimental study may differ from the clinical condition, and this is a limitation of the study. Therefore, further investigation including the effects of delayed VNS treatment on heatstroke is required.

In conclusion, we demonstrated in a rat model of heatstroke that electrical VNS suppressed systemic inflammation occurring secondary to heat stress and eventually improved survival. The present study defined the role of the cholinergic anti-inflammatory pathway in the pathophysiology of heatstroke. These findings may provide a novel therapeutic strategy using electrical VNS to combat severe heatstroke in the critical care setting.

## References

[1] Kondo M, Honda Y, Ono M (2011) Growing concern about heatstroke this summer in Japan after Fukushima nuclear disaster. Environ Health Prev Med 16: 279–280. 21833691 10.1007/s12199-011-0228-8 PMC3156841

[2] Bouchama A, Knochel JP (2002) Heat stroke. N Engl J Med 346: 1978–1988. 12075060 10.1056/NEJMra011089

[3] Leon LR, Helwig BG (2010) Heat stroke: role of the systemic inflammatory response. J Appl Physiol 109: 1980–1988. 20522730 10.1152/japplphysiol.00301.2010

[4] Bouchama A (1995) Heatstroke: a new look at an ancient disease. Intensive Care Med 21: 623–625. 8522663 10.1007/BF01711537

[5] Borovikova LV, Ivanova S, Zhang M, Yang H, Botchkina GI, et al (2000) Vagus nerve stimulation attenuates the systemic inflammatory response to endotoxin. Nature 405: 458–462. 10839541 10.1038/35013070

[6] Wang H, Yu M, Ochani M, Amella CA, Tanovic M, et al (2003) Nicotinic acetylcholine receptor alpha7 subunit is an essential regulator of inflammation. Nature 421: 384–388. 12508119 10.1038/nature01339

[7] Guarini S, Altavilla D, Cainazzo MM, Giuliani D, Bigiani A, et al (2003) Efferent vagal fibre stimulation blunts nuclear factor-kappaB activation and protects against hypovolemic hemorrhagic shock. Circulation 107: 1189–1194. 12615800 10.1161/01.cir.0000050627.90734.ed

[8] Cai B, Chen F, Ji Y, Kiss L, de Jonge WJ, et al (2009) Alpha7 cholinergic-agonist prevents systemic inflammation and improves survival during resuscitation. J Cell Mol Med 13: 3774–3785. 19602049 10.1111/j.1582-4934.2008.00550.x PMC3046874

[9] Bernik TR, Friedman SG, Ochani M, DiRaimo R, Susarla S, et al (2002) Cholinergic antiinflammatory pathway inhibition of tumor necrosis factor during ischemia reperfusion. J Vasc Surg 36: 1231–1236. 12469056 10.1067/mva.2002.129643

[10] Altavilla D, Guarini S, Bitto A, Mioni C, Giuliani D, et al (2006) Activation of the cholinergic anti-inflammatory pathway reduces NF-kappab activation, blunts TNF-alpha production, and protects againts splanchic artery occlusion shock. Shock 25: 500–506. 16680015 10.1097/01.shk.0000209539.91553.82

[11] Bernik TR, Friedman SG, Ochani M, DiRaimo R, Ulloa L, et al (2002) Pharmacological stimulation of the cholinergic antiinflammatory pathway. J Exp Med 195: 781–788. 11901203 10.1084/jem.20011714 PMC2193742

[12] Wang H, Liao H, Ochani M, Justiniani M, Lin X, et al (2004) Cholinergic agonists inhibit HMGB1 release and improve survival in experimental sepsis. Nat Med 10: 1216–1221. 15502843 10.1038/nm1124

[13] van Westerloo DJ, Giebelen IA, Florquin S, Daalhuisen J, Bruno MJ, et al (2005) The cholinergic anti-inflammatory pathway regulates the host response during septic peritonitis. J Infect Dis 191: 2138–2148. 15898001 10.1086/430323

[14] Chang CK, Chang CP, Chiu WT, Lin MT (2006) Prevention and repair of circulatory shock and cerebral ischemia/injury by various agents in experimental heatstroke. Curr Med Chem 13: 3145–3154. 17168703 10.2174/092986706778742945

[15] Sharma HS (2005) Heat-related deaths are largely due to brain damage. Indian J Med Res 121: 621–623. 15937362

[16] Hsiao SH, Chang CP, Chiu TH, Lin MT (2007) Resuscitation from experimental heatstroke by brain cooling therapy. Resuscitation 73: 437–445. 17300862 10.1016/j.resuscitation.2006.11.003

[17] Shen KH, Lin CH, Chang HK, Chen WC, Chen SH (2008) Premarin can act via estrogen receptors to rescue mice from heatstroke-induced lethality. Shock 30: 668–674. 18496235 10.1097/SHK.0b013e31817538cb

[18] Hagiwara S, Iwasaka H, Matsumoto S, Hasegawa A, Yasuda N, et al (2010) In vivo and in vitro effects of the anticoagulant, thrombomodulin, on the inflammatory response in rodent models. Shock 33: 282–288. 19536047 10.1097/SHK.0b013e3181b0ef7b

[19] Hagiwara S, Iwasaka H, Shingu C, Matsumoto S, Uchida T, et al (2010) High-dose antithrombin III prevents heat stroke by attenuating systemic inflammation in rats. Inflamm Res 59: 511–518. 20047080 10.1007/s00011-009-0155-y

[20] Hagiwara S, Iwasaka H, Shingu C, Matsumoto S, Uchida T, et al (2011) Danaparoid sodium attenuates the effects of heat stress. J Surg Res 171: 762–768. 20673918 10.1016/j.jss.2010.05.008

[21] Nybo L (2007) Exercise and heat stress: cerebral challenges and consequences. Prog Brain Res 162: 29–43. 17645913 10.1016/S0079-6123(06)62003-7

[22] Bouchama A, Kunzelmann C, Dehbi M, Kwaasi A, Eldali A, et al (2008) Recombinant activated protein C attenuates endothelial injury and inhibits procoagulant microparticles release in baboon heatstroke. Arterioscler Thromb Vasc Biol 28: 1318–1325. 18451327 10.1161/ATVBAHA.107.161737

[23] Chen CC, Chen ZC, Lin MT, Hsu CC (2009) Activated protein C improves heatstroke outcomes through restoration of normal hypothalamic and thermoregulatory function. Am J Med Sci 338: 382–387. 19773641 10.1097/MAJ.0b013e3181b272ae

[24] Bouchama A, Parhar RS, el-Yazigi A, Sheth K, al-Sedairy S (1991) Endotoxemia and release of tumor necrosis factor and interleukin 1 alpha in acute heatstroke. J Appl Physiol 70: 2640–2644. 1885459 10.1152/jappl.1991.70.6.2640

[25] Tracey KJ (2002) The inflammatory reflex. Nature 420: 853–859. 12490958 10.1038/nature01321

[26] Niederbichler AD, Papst S, Claassen L, Jokuszies A, Steinstraesser L, et al (2009) Burn-induced organ dysfunction: vagus nerve stimulation attenuates organ and serum cytokine levels. Burns 35: 783–789. 19482432 10.1016/j.burns.2008.08.023

[27] Song XM, Li JG, Wang YL, Liang H, Huang Y, et al (2010) Effect of vagus nerve stimulation on thermal injury in rats. Burns 36: 75–81. 19646819 10.1016/j.burns.2009.02.002

[28] Bansal V, Costantini T, Ryu SY, Peterson C, Loomis W, et al (2010) Stimulating the central nervous system to prevent intestinal dysfunction after traumatic brain injury. J Trauma 68: 1059–1064. 20453760 10.1097/TA.0b013e3181d87373 PMC4251579

[29] Roberts GT, Ghebeh H, Chishti MA, Al-Mohanna F, El-Sayed R, et al (2008) Microvascular injury, thrombosis, inflammation, and apoptosis in the pathogenesis of heatstroke: a study in baboon model. Arterioscler Thromb Vasc Biol 28: 1130–1136. 18388331 10.1161/ATVBAHA.107.158709

[30] Huang H, Lavoie-Lamoureux A, Lavoie JP (2009) Cholinergic stimulation attenuates the IL-4 induced expression of E-selectin and vascular endothelial growth factor by equine pulmonary artery endothelial cells. Vet Immunol Immunopathol 132: 116–121. 19501920 10.1016/j.vetimm.2009.05.003

[31] Saeed RW, Varma S, Peng-Nemeroff T, Sherry B, Balakhaneh D, et al (2005) Cholinergic stimulation blocks endothelial cell activation and leukocyte recruitment during inflammation. J Exp Med 201: 1113–1123. 15809354 10.1084/jem.20040463 PMC2213139

[32] Wessler I, Kirkpatrick CJ (2008) Acetylcholine beyond neurons: the non-neuronal cholinergic system in humans. Br J Pharmacol 154: 1558–1571. 18500366 10.1038/bjp.2008.185 PMC2518461

[33] Pohanka M, Snopkova S, Havlickova K, Bostik P, Sinkorova Z, et al (2011) Macrophage-assisted inflammation and pharmacological regulation of the cholinergic anti-inflammatory pathway. Curr Med Chem 18: 539–551. 21143112 10.2174/092986711794480140

[34] Huston JM, Ochani M, Rosas-Ballina M, Liao H, Ochani K, et al (2006) Splenectomy inactivates the cholinergic antiinflammatory pathway during lethal endotoxemia and polymicrobial sepsis. J Exp Med 203: 1623–1628. 16785311 10.1084/jem.20052362 PMC2118357

[35] Swirski FK, Nahrendorf M, Etzrodt M, Wildgruber M, Cortez-Retamozo V, et al (2009) Identification of splenic reservoir monocytes and their deployment to inflammatory sites. Science 325: 612–616. 19644120 10.1126/science.1175202 PMC2803111

